# Simultaneous occurrence of epidural, subdural, and subarachnoid hemorrhages in the spinal canal: a rare case report

**DOI:** 10.1007/s00234-025-03576-3

**Published:** 2025-03-28

**Authors:** Arda Çolakoğlu, Kerim Aslan, Barış Genç, Lütfi İncesu

**Affiliations:** Department of Radiology, Ondokuz Mayıs University Faculty of Medicine, Samsun, Turkey

**Keywords:** Magnetic resonance imaging, Spine, Hemorrhage, Hematoma

## Abstract

Simultaneous spinal epidural, subdural, and subarachnoid hemorrhages are exceedingly rare, with no prior documented case of all three occurring simultaneously in the literature. We present the case of a 59-year-old woman who developed acute lower back pain and progressive weakness in her lower extremities. MRI revealed three distinct types of hemorrhages: an epidural hematoma extending from T12 to the lumbar spine, a subdural hematoma between T11 and S1, and a subarachnoid hemorrhage involving the cauda equina. Surgical decompression was performed, leading to significant clinical improvement. This case underscores the critical role of MRI in the diagnosis of complex spinal hematomas and highlights the importance of prompt surgical intervention to prevent further neurological deterioration. Our report contributes valuable insights into the management of multifaceted spinal hemorrhages, emphasizing the need for early recognition and treatment in such rare presentations.

**Supplementary Information:**

The online version contains supplementary material available at 10.1007/s00234-025-03576-3.

## Introduction

Spinal hematomas, though rare, are critical neurological emergencies that can present as epidural, subdural, subarachnoid or intramedullary hemorrhages, each associated with distinct causes and clinical outcomes. Epidural spinal hematomas most commonly arise from ruptured vessels in the epidural space, often linked to trauma, coagulopathy, or iatrogenic causes [1, 2]. Subdural hemorrhages are typically associated with similar causes but it is even rarer, while subarachnoid hemorrhages are usually due to ruptured aneurysms or vascular malformations [3]. The simultaneous occurrence of different types of hemorrhages within the spinal canal is extremely uncommon, with no known cases in the literature reporting all three extramedullary hemorrhage types occurring simultaneously. Individually, each type can cause significant neurological dysfunction by compressing spinal structures, but their co-occurrence poses a unique diagnostic challenge.

Magnetic resonance imaging (MRI) is the gold standard for diagnosing spinal hemorrhages. MRI not only confirms the presence of bleeding but also helps delineate the anatomical compartments affected, allowing clear differentiation between epidural, subdural, and subarachnoid hemorrhages [4]. In this case, spinal MRI was crucial in identifying the rare coexistence of all three hemorrhage types within the spinal canal. The aim of this case report is to emphasize the importance of MRI in the diagnosis and management of an unprecedented spinal hematoma in multiple compartments, contributing to the limited understanding of multiple concurrent spinal hemorrhages.

## Case report

A 59-year-old woman was admitted to our center with complaints of severe lower back pain and progressive weakness in her lower extremities over the past two days. She stated that she had been able to mobilize with support the day before admission, but on the day of admission, she was unable to move her legs at all. There was no history of trauma. Her known medical conditions were hypertension and arrhythmia. On examination, she had diffuse pain and numbness in both lower extremities. There was no urinary or fecal incontinence. Her Glasgow Coma Score was 15. Muscle strength was 1/5 in both lower extremities. Bilateral deep tendon reflexes were normal, but bilateral clonus and Babinski reflexes were positive.

**Fig. 1:**
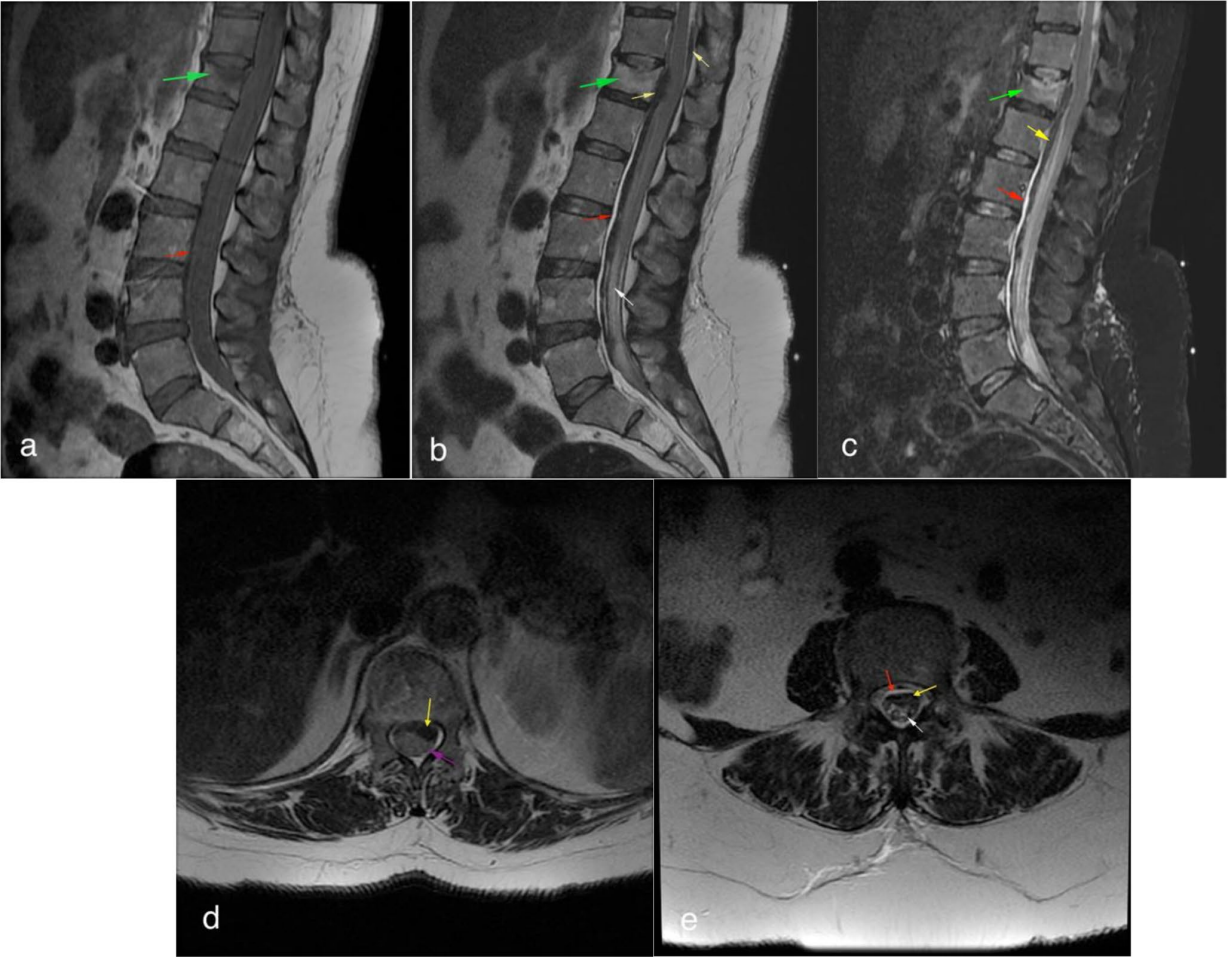
An acute compression fracture of the T12 vertebral body with 40% height loss is observed (**a**-**c**, green arrow). At the lumbar level, a 1 mm thick epidural hematoma that is isointense on T1 weighted images (**a**) and hyperintense on T2 weighted and short tau inversion recovery (STIR) images (**b**, **c**) is present in the anterior epidural space, where the anterior epidural fatty tissue is not visible (red arrow). A subdural hematoma, isointense on T1 and hypointense on T2, extends from the T11 to S1 levels, with a maximum thickness of 7.5 mm in the anterior subdural space and involvement of the posterior subdural space as well (**b**-**e**, yellow arrow). On axial T2 images (**d**), the hematomas narrow the spinal canal and compress the spinal cord at the T12 level (purple arrow). Additionally, T2 axial images at the lumbar level (**e**) demonstrate hyperintense subarachnoid hemorrhage within the spinal canal, displacing the cauda equina fibers (white arrow)

The patient underwent a thoracolumbar spinal MRI. The MRI revealed an acute compression fracture of the superior endplate of the T12 vertebral body with a 40% height loss. Beginning at this level and extending inferiorly along the lumbar spine in the anterior epidural space, there was a 1 mm thick T1 isointense, T2 hyperintense epidural hematoma in the hyperacute stage. Additionally, a T1 isointense, T2 hypointense subdural hematoma in the acute stage was observed, extending between the T11 and S1 levels in the anterior and posterior subdural space, with a maximum thickness of 7.5 mm measured at the T12 level. Due to these extra axial hematomas, the spinal canal was narrowed, most prominently at the T12 level, causing cord compression. Furthermore, T2 hyperintense subarachnoid hemorrhage was visible between the cauda equina fibers along the lumbar spinal canal, resulting in aggregation of the fibers (Fig. 1).

Based on these findings, the decision was made to proceed with surgery by the neurosurgery team. Bilateral transpedicular fixation screws were placed at the T11, T12, and L1 levels. T12 left hemilaminectomy was then performed. It was determined that the neural root was detached, and the bleeding occurred from there. The root was decompressed, and Surgicel was applied for hemostasis. The operation was completed without complications.

**Fig. 2:**
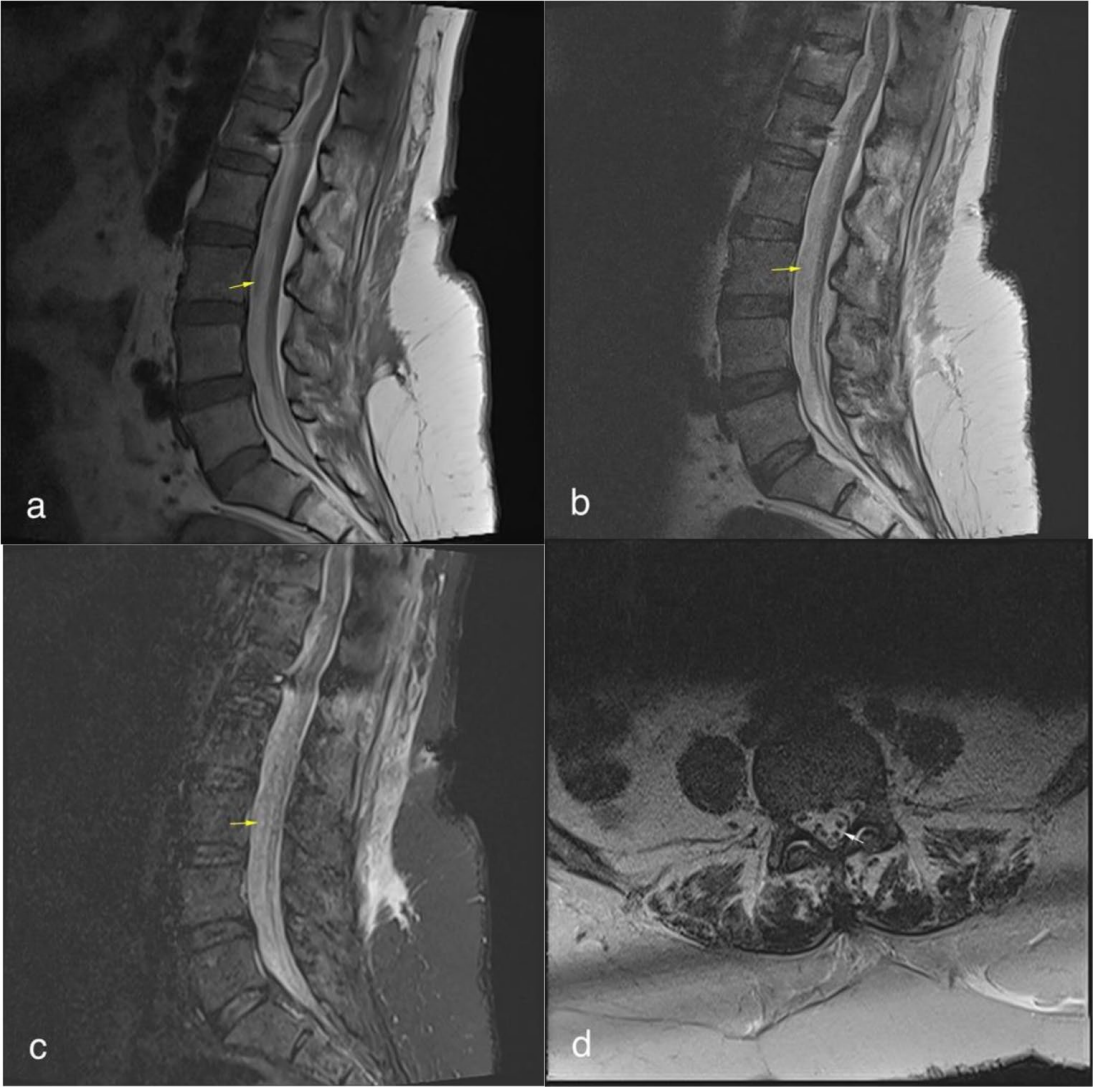
Bilateral transpedicular fixation screws are present at the T11-L1 level, along with postoperative changes in the posterior subcutaneous soft tissues. A 7 mm thick subdural hematoma, hyperintense on both T1 weighted (**a**), T2 weighted, and short tau inversion recovery (STIR) (**b**, **c**) images, is observed in the anterior subdural space from T11 to S1 levels (yellow arrow). Clumping of the cauda equina fibers is noted, likely due to a previous subarachnoid hemorrhage (**d**, white arrow)

Postoperative MRI taken one week later revealed postoperative changes in the subcutaneous soft tissues at the thoracolumbar level and bilateral transpedicular fixation screws on T11-L1 levels. A 6 mm thick T1 and T2 hyperintense subdural collection, consistent with a late subacute stage hematoma, was observed in the anterior subdural space from the T12 to the S1 level. Aggregation of the cauda equina fibers, consistent with previous subarachnoid hemorrhage, was also noted. The epidural hematoma seen in the anterior epidural space in the prior examination was no longer visible (Fig. 2). The patient’s symptoms improved during the postoperative period, and she was discharged two weeks after surgery.

## Discussion

MRI plays a pivotal role in diagnosing spinal hemorrhages. MRI provides detailed information about the anatomical location of the hemorrhage, the affected structures, and the stage of the hemorrhage [2]. The signal characteristics of the hematoma help determine whether the hemorrhage is in the hyperacute, acute, early/late subacute, or chronic stage. Due to MRI’s high soft tissue resolution, the anatomical compartment where the hemorrhage occurs, the potential cause, and the affected structures can be accurately visualized [2, 5].

Epidural hematomas are located in the epidural space between the dura mater and the vertebrae. They create external compression on the thecal sac and displace the dura mater toward the spinal cord. The loss of normal epidural fat signal is an important indicator of an epidural hematoma [2, 5]. In our case, the anterior epidural fatty tissue could not be distinguished due to the presence of the epidural hematoma.

Subdural hematomas occur in the potential space between the dura mater and the arachnoid membrane. In subdural hematomas, the epidural fatty tissue is preserved, and inward displacement of the dura mater is not observed [2, 5]. In our case, MRI clearly demonstrated the subdural hematoma, which narrowed the spinal canal and exerted compression on the spinal cord.

Subarachnoid hemorrhages occur into the CSF between the arachnoid membrane and the pia mater. Subarachnoid hemorrhage spreads longitudinally due to CSF flow, and hematoma formation is typically not observed [6]. T2 hyperintense hemorrhage or clot formation may be seen around the spinal cord and between the cauda equina fibers. Aggregation of cauda equina fibers may occur at the lumbar level due to compression [2, 5]. In our case, MRI clearly demonstrated subarachnoid hemorrhage leading to aggregation of the cauda equina fibers.

In our case, spinal MRI revealed clear separation of the hemorrhages into three compartments, as well as cord compression and clumping of the cauda equina fibers in the lumbar region. The rapid diagnosis facilitated by spinal MRI allowed for timely surgical intervention, which was crucial in preventing further neurological decline.

## Conclusion

This case report highlights the unique and complex presentation of simultaneous epidural, subdural, and subarachnoid hemorrhages within the spinal canal, emphasizing the critical role of MRI in their diagnosis and management. The unique coexistence of these hemorrhages presented significant challenges, yet timely MRI imaging and surgical intervention were pivotal in preventing further neurological decline. Postoperative improvements in the patient’s symptoms underline the importance of early identification and prompt treatment in cases of spinal hematomas.

## Electronic supplementary material

Below is the link to the electronic supplementary material.

Supplementary Material 1

Supplementary Material 2

Supplementary Material 3

Supplementary Material 4

Supplementary Material 5

Supplementary Material 6

Supplementary Material 7

Supplementary Material 8

Supplementary Material 9
