# Supplementary figures and images for: Simultaneous occurrence of epidural, subdural, and subarachnoid hemorrhages in the spinal canal: a rare case report
Source: Neuroradiology. 2025 Mar 28;67(4):1091–4. doi: 10.1007/s00234-025-03576-3 (PMC12041143; doi:10.1007/s00234-025-03576-3)

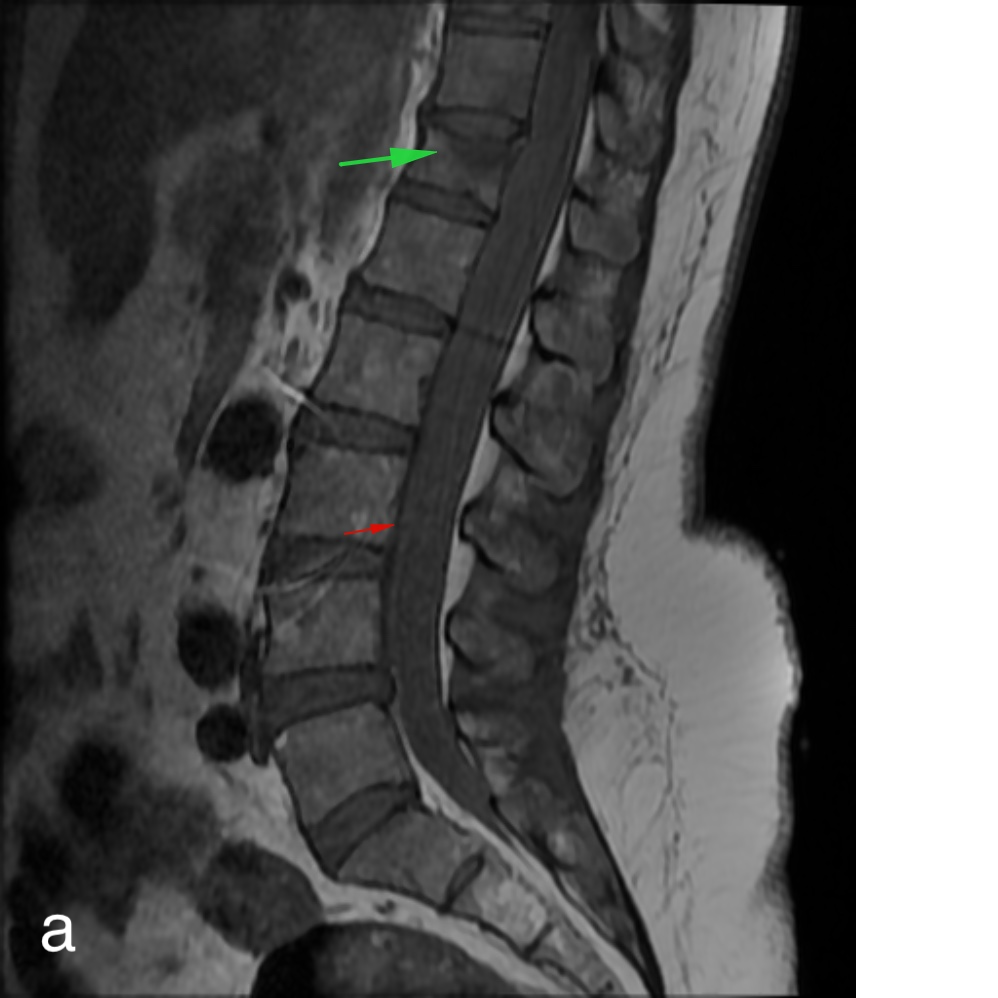

Supplement: Supplementary file 1 — Supplementary Material 1 [file 234_2025_3576_MOESM1_ESM.png]

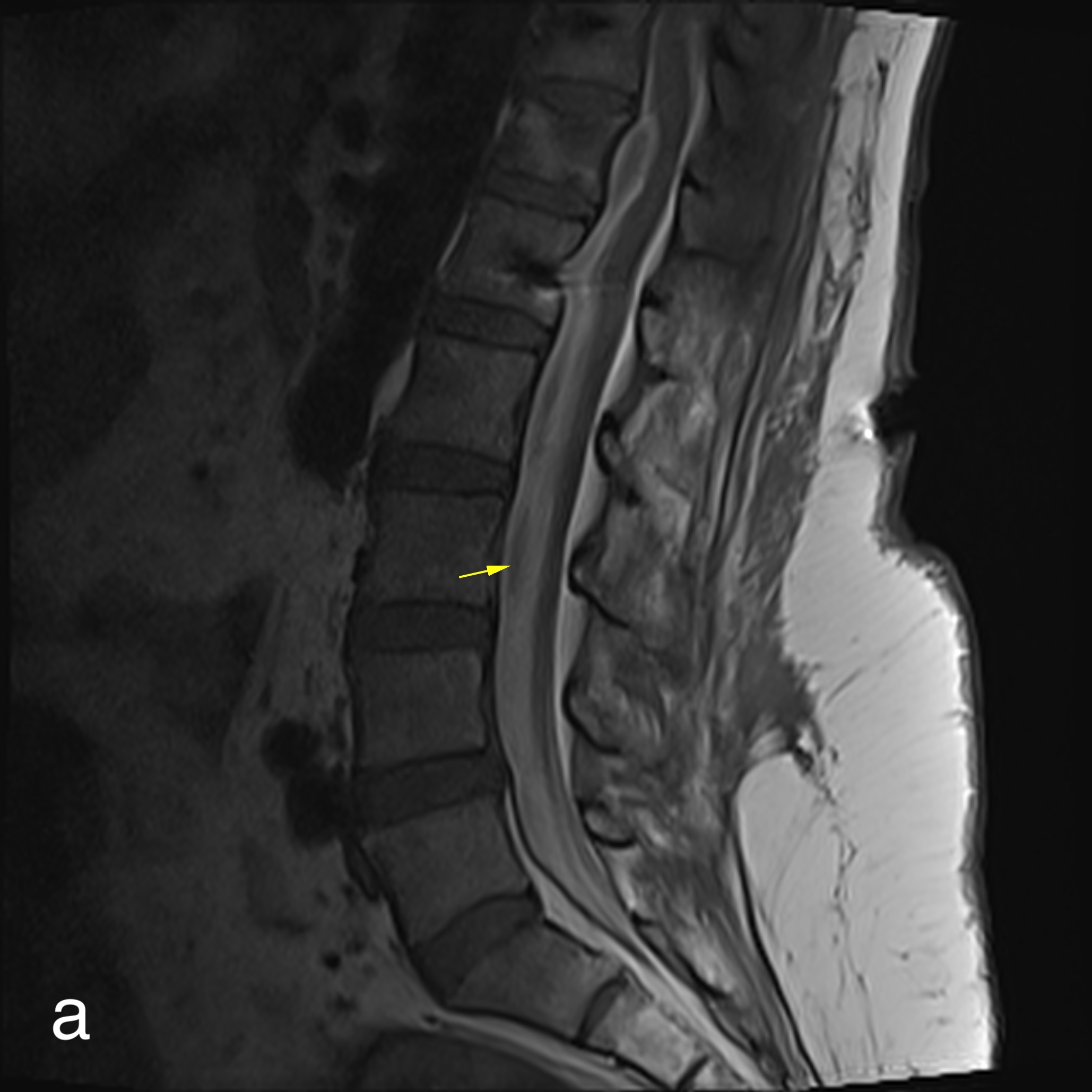

Supplement: Supplementary file 2 — Supplementary Material 2 [file 234_2025_3576_MOESM2_ESM.jpg]

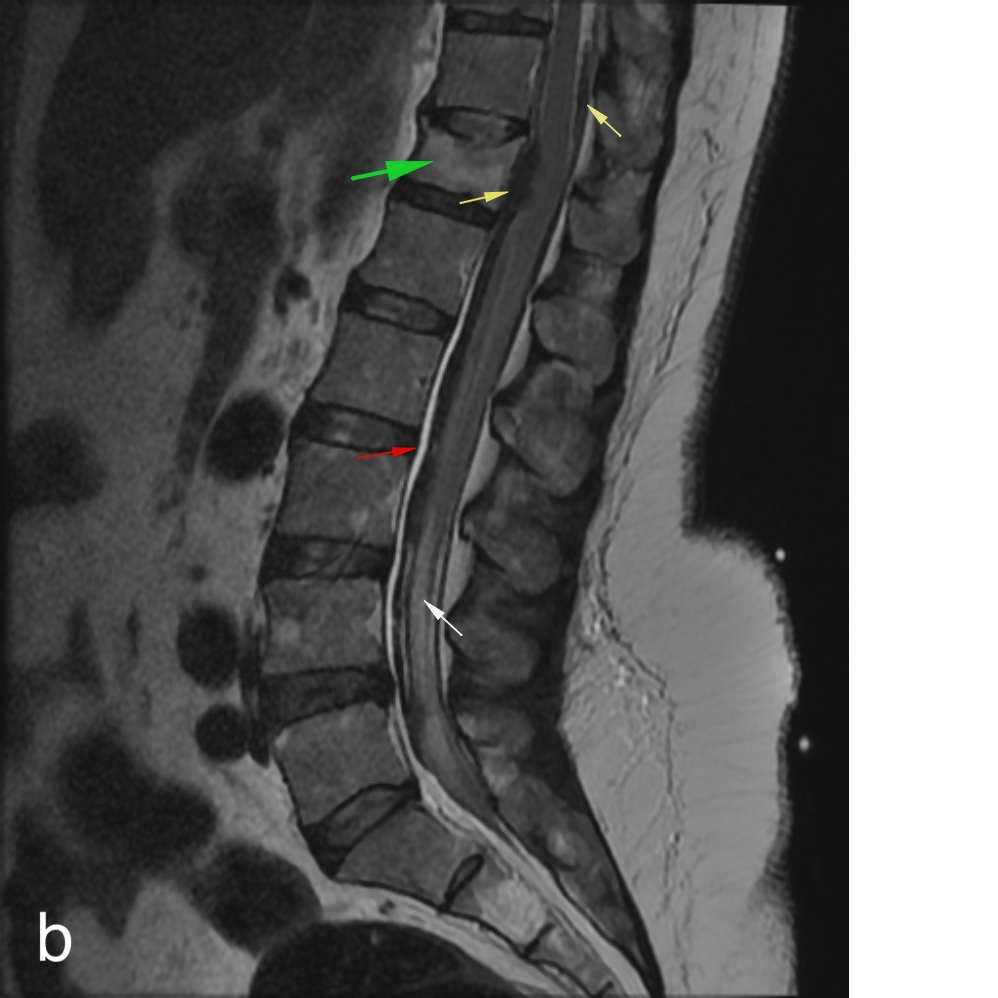

Supplement: Supplementary file 3 — Supplementary Material 3 [file 234_2025_3576_MOESM3_ESM.png]

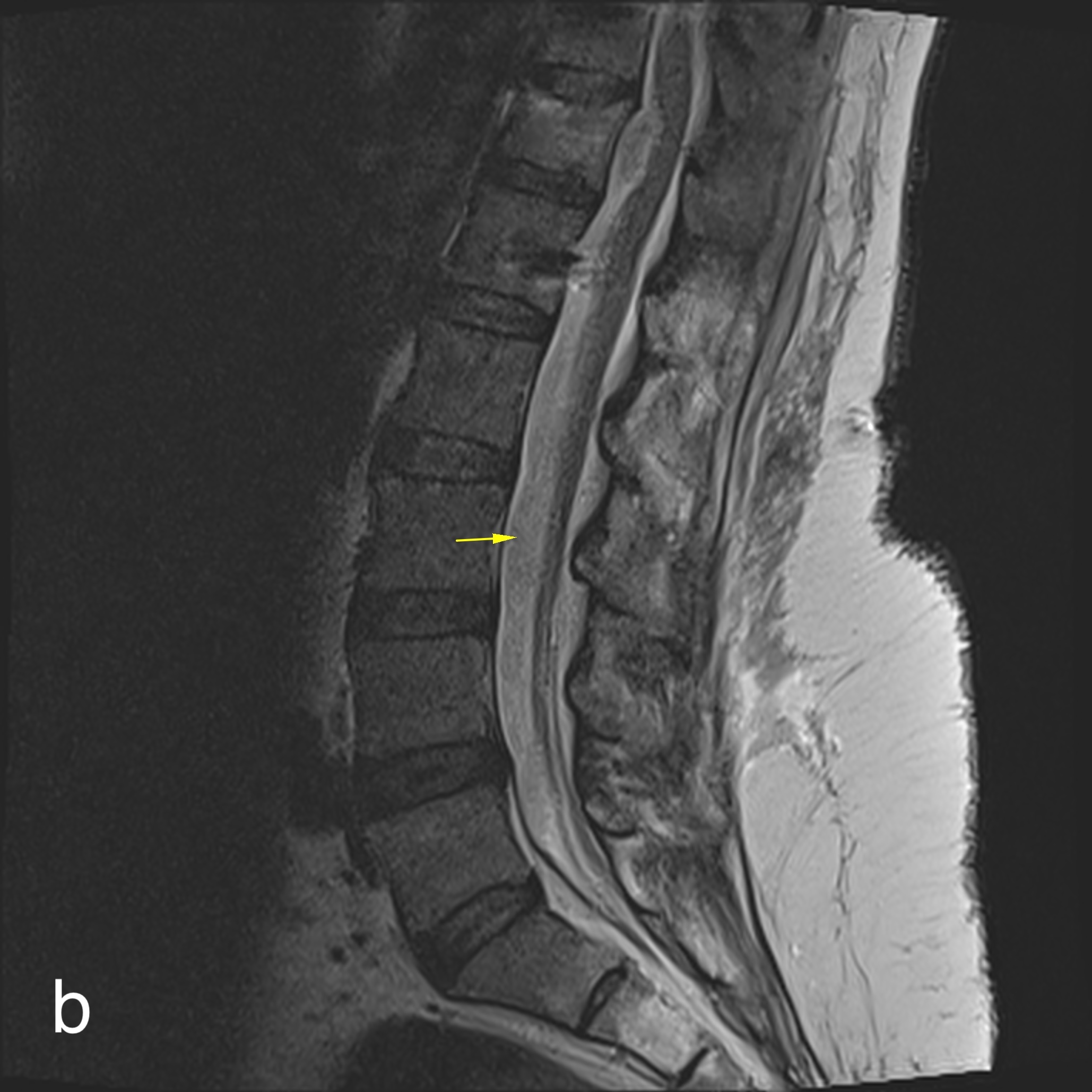

Supplement: Supplementary file 4 — Supplementary Material 4 [file 234_2025_3576_MOESM4_ESM.jpg]

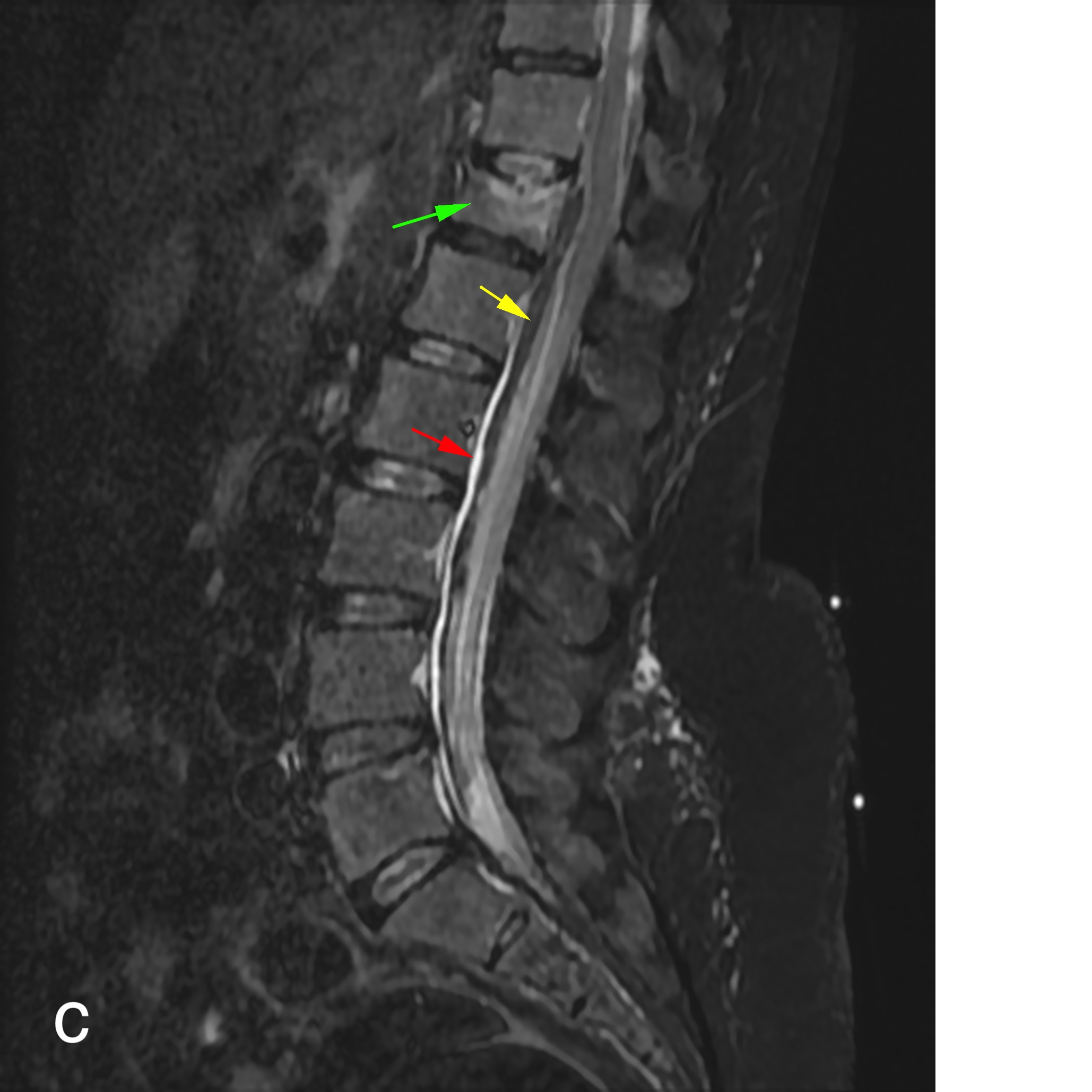

Supplement: Supplementary file 5 — Supplementary Material 5 [file 234_2025_3576_MOESM5_ESM.png]

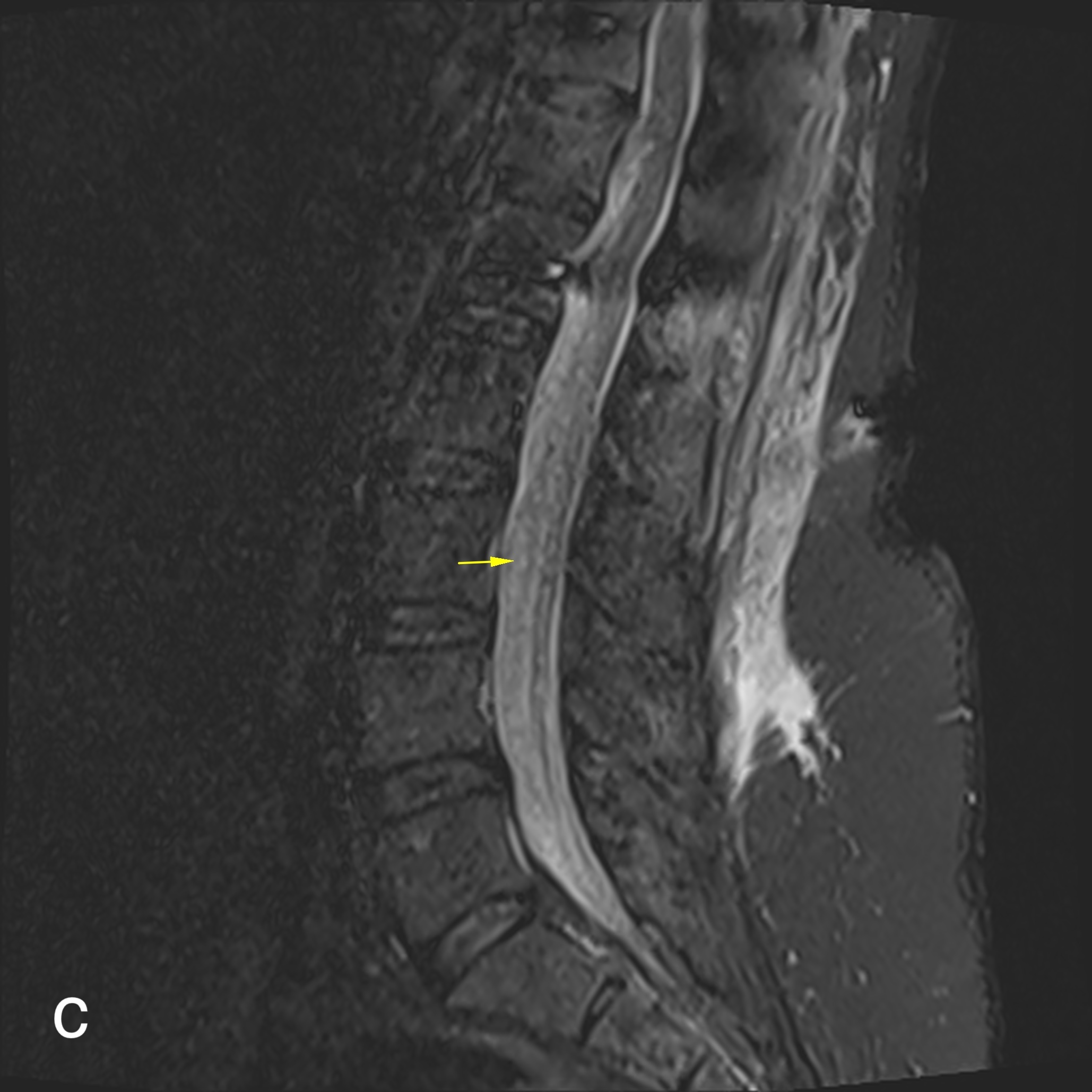

Supplement: Supplementary file 6 — Supplementary Material 6 [file 234_2025_3576_MOESM6_ESM.jpg]

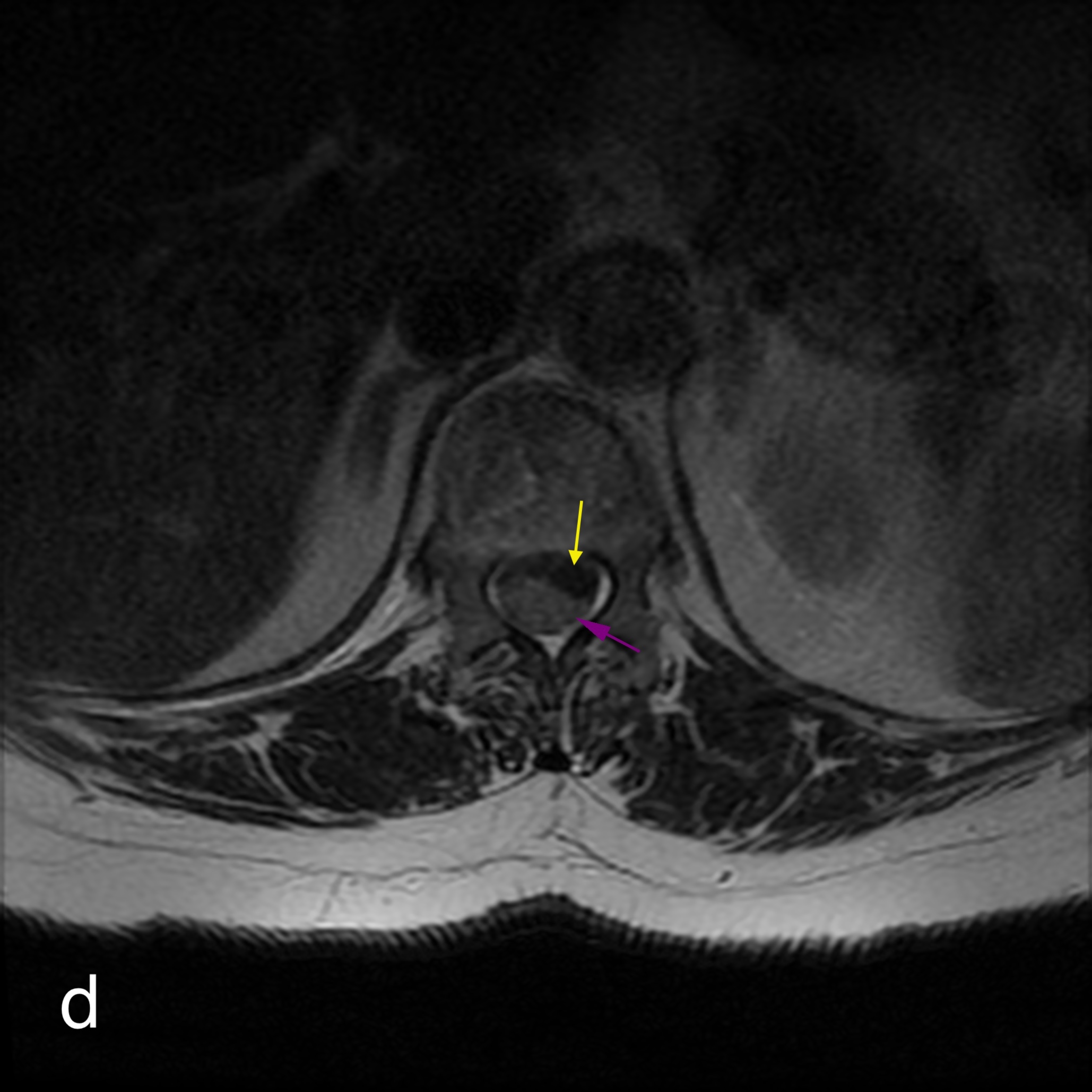

Supplement: Supplementary file 7 — Supplementary Material 7 [file 234_2025_3576_MOESM7_ESM.jpg]

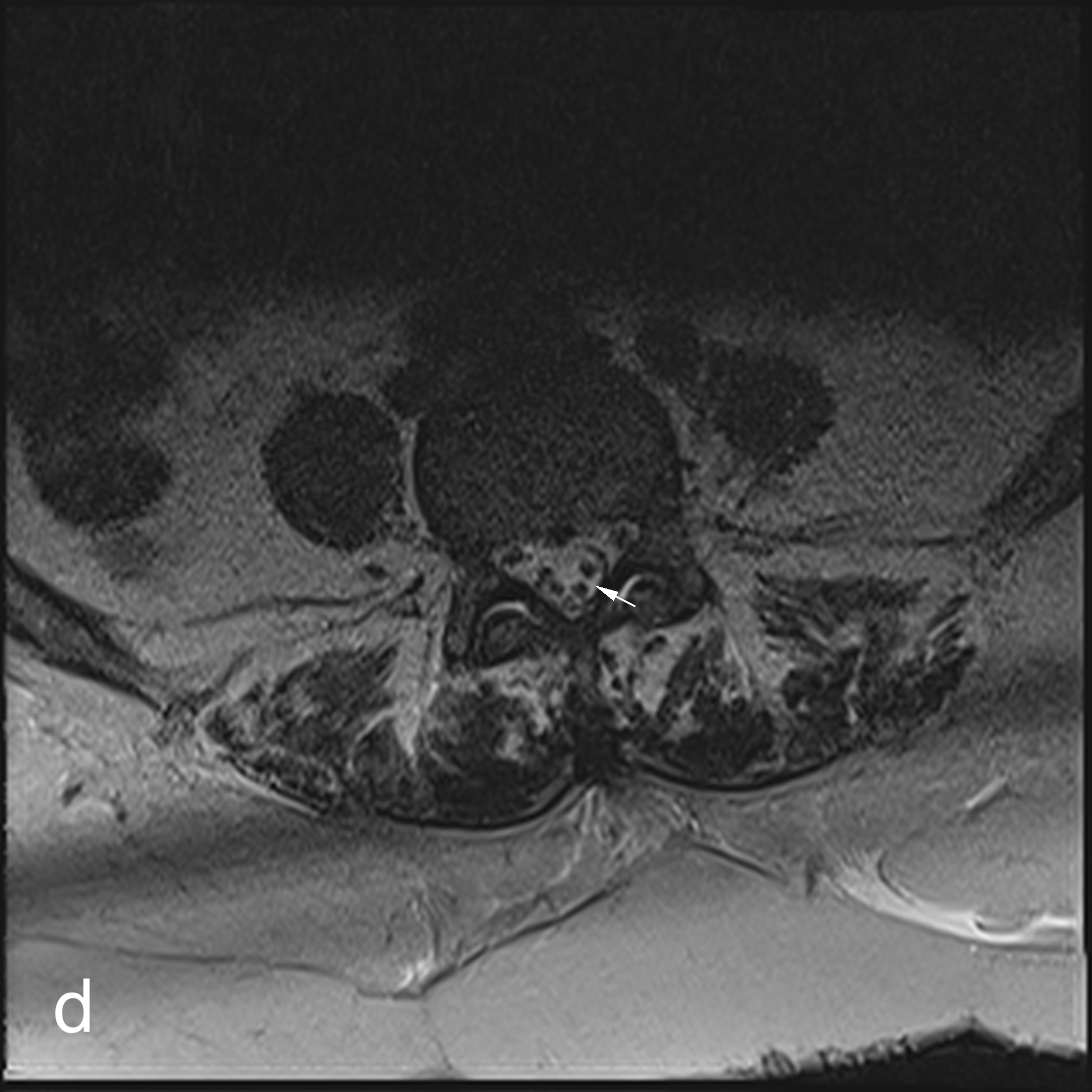

Supplement: Supplementary file 8 — Supplementary Material 8 [file 234_2025_3576_MOESM8_ESM.jpg]

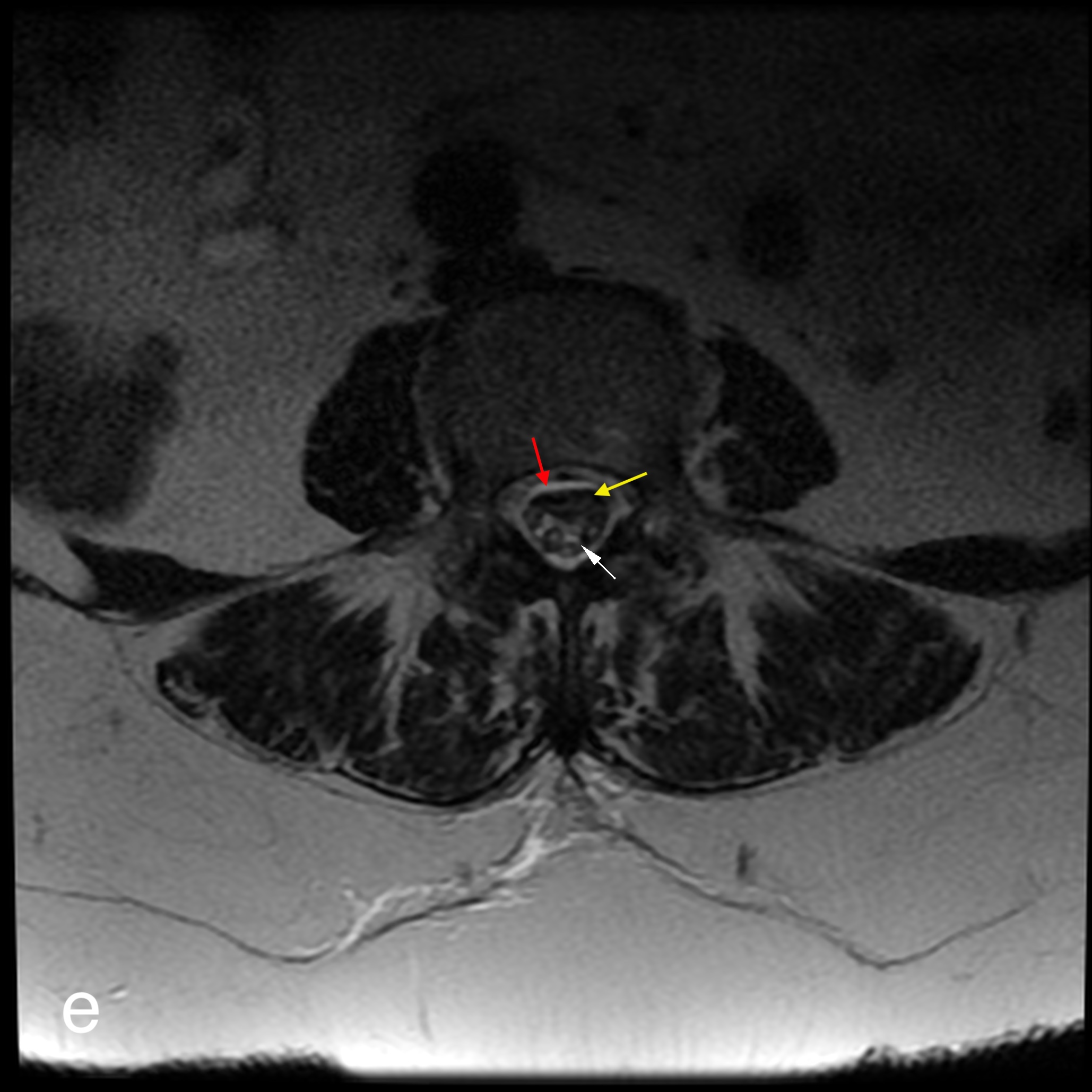

Supplement: Supplementary file 9 — Supplementary Material 9 [file 234_2025_3576_MOESM9_ESM.jpg]
